# Development of a Lethal Intranasal Exposure Model of Ebola Virus in the Cynomolgus Macaque

**DOI:** 10.3390/v9110319

**Published:** 2017-10-29

**Authors:** Kendra J. Alfson, Laura E. Avena, Gabriella Worwa, Ricardo Carrion, Anthony Griffiths

**Affiliations:** 1 Department of Virology and Immunology, Texas Biomedical Research Institute, San Antonio, TX 78227, USA; kalfson@txbiomed.org (K.J.A.); lavena@txbiomed.org (L.E.A.); gworwa@txbiomed.org (G.W.); rcarrion@TxBiomed.org (R.C.J.); 2 Graduate School of Biomedical Sciences, University of Texas Health Science Center at San Antonio, 7703 Floyd Curl Drive, San Antonio, TX 78229, USA

**Keywords:** Ebola virus, intranasal, animal model

## Abstract

Ebola virus (EBOV) is a filovirus that can cause Ebola virus disease (EVD). No approved vaccines or therapies exist for filovirus infections, despite an urgent need. The development and testing of effective countermeasures against EBOV requires use of animal models and a thorough understanding of how the model aligns with EVD in humans. The majority of published studies report outcomes of parenteral exposures for emulating needle stick transmission. However, based on data from EVD outbreaks, close contact exposures to infected bodily fluid seems to be one of the primary routes of EBOV transmission. Thus, further work is needed to develop models that represent mucosal exposure. To characterize the outcome of mucosal exposure to EBOV, cynomolgus macaques were exposed to EBOV via intranasal (IN) route using the LMA^®^ mucosal atomization device (LMA^®^ MAD). For comparison, four non-human primates (NHPs) were exposed to EBOV via intramuscular (IM) route. This IN exposure model was uniformly lethal and correlated with a statistically significant delay in time to death when compared to exposure via the IM route. This more closely reflects the timeframes observed in human infections. An IN model of exposure offers an attractive alternative to other models as it can offer insight into the consequences of exposure via a mucosal surface and allows for screening countermeasures via a different exposure route.

## 1. Introduction

Ebola virus (EBOV) is a filovirus that can cause Ebola virus disease (EVD), commonly referred to as Ebola hemorrhagic fever prior to the 2014–2016 western Africa outbreak [1]. Filoviruses are important biothreats due to the high case fatality rates combined with potential for spread to new regions of the world, new hosts, and even new modes of infection [1,2,3,4]. There are no licensed countermeasures for filovirus infection and the western Africa outbreak emphasizes the urgency for vaccines and therapeutics [1].

Disease modeling and pathogenesis studies rely heavily on well-characterized animal models. Furthermore, as determination of filovirus-countermeasure efficacy in humans is unfeasible and unethical, the US Food and Drug Administration may employ the Animal Efficacy Rule (21 CFR 314.600 and 601.90) to license countermeasures relying on animal efficacy data. [5,6]. Consequently, the development and testing of effective countermeasures against EBOV will require the development of well-characterized animal models and a thorough understanding of those models. Additionally, the unprecedented western Africa outbreak has led public health officials and scientists to recognize the critical importance of understanding EBOV pathogenesis and virulence [7,8].

Several animal models exist to study EBOV, including mice [9], guinea pigs [10], ferrets [11], and nonhuman primates (NHP) such as rhesus and cynomolgus macaques (*Macaca mulatta* and *Macaca fascicularis*, respectively). However, immunocompetent rodent models require the use of adapted virus and often fail to recapitulate many attributes of the human disease [12]. In contrast, the animals used in NHP models are susceptible to infection with wild-type non-adapted viruses and exhibit disease attributes and pathology similar to that seen in humans with EVD. Consequently, NHPs are the animal model of choice for studying EBOV [12,13].

Based on data from previous EVD outbreaks and the 2014–2016 western Africa outbreak, close-contact exposures to infected bodily fluid seem to be one of the primary routes of EBOV transmission [7,14,15,16]. However, the majority of published studies in NHPs report lethal outcomes after parenteral routes of exposure (usually intramuscular (IM) to mimic needle stick) [13,15,17], although a few studies have described lethal infection via oral or conjunctival [18,19], and aerosol [20,21] routes. Thus, further work is needed to develop and characterize models that represent the bodily fluid transmission seen during human EVD outbreaks.

An intranasal route of exposure can be used to evaluate mucosal exposures associated with direct facial or nostril contact with EBOV-contaminated sprays or droplets. The LMA^®^ Mucosal Atomization Device (MAD) (Teleflex) allows for intranasal (IN) delivery of atomized particles 30 - 100 microns in size, which thus model droplet transmission and are most likely to target the surfaces in the upper respiratory tract [22]. The LMA^®^ MAD was developed for safe and efficient drug delivery and is used to administer drugs that are United States Food and Drug Administration (U.S. FDA) approved for IN delivery. Herein, we describe a lethal IN model of EBOV exposure in cynomologus macaques and compare the model to the more common lethal IM exposure model.

## 2. Materials and Methods

### 2.1. Ethics Statement

Animal research was conducted under Institutional Animal Care and Use Committee (IACUC)-approved protocols (IACUC numbers 1381, 20 August 2013; 1532, 12 April 2016 and 1529, 24 March 2016) in compliance with the Animal Welfare Act and other federal statutes and regulations relating to animals and experiments involving animals. Texas Biomedical Research Institute (TBRI) is accredited by the Association for Assessment and Accreditation of Laboratory Animal Care International and adheres to principles stated in the 8th Edition of the Guide for the Care and Use of Laboratory Animals, National Research Council [23]. Euthanasia criteria were developed to minimize undue pain and distress and animals were euthanized with an intravenous overdose of sodium pentobarbital. Clinical scores for the animals were reported to the responsible veterinarian, and euthanasia was approved when scores indicated that an animal was terminally ill. Additional euthanasia criteria included a combination of severe petechia or bleeding from any orifice, temperature change of greater than 5 °F from baseline, moderate to severe depression (determined by evaluating responsiveness), respiratory distress, or clinically-significant increases in γ-glutamyltransferase (GGT), alanine aminotransferase (ALT), alkaline phosphatase (ALP), or blood urea nitrogen (BUN) [24].

### 2.2. Cells and Virus

BEI-sourced Vero E6 cells (African green monkey kidney cell origin) were grown in Minimum Essential Media (MEM; Gibco, Grand Island, NY, USA) containing 2 mM L-glutamine (Gibco, Grand Island, NY, USA) and 1 mM sodium pyruvate (Gibco, Grand Island, NY, USA) (henceforth referred to as normal growth media) with 10% heat-inactivated fetal calf serum (FCS; Gibco, Grand Island, NY, USA) at 37 °C with 5% CO_2_ [25,26]. Ebola virus (Kikwit variant), passage number 2 on Vero E6 cells (Ebola virus H.sapiens-tc/COD/1995/Kikwit-9510621, species *Zaire ebolavirus*) was acquired from Dr. T. Ksiazek, University of Texas Medical Branch [25,26]. This stock was then passaged one time in Vero E6 cells at a multiplicity of infection of 0.001 to generate a passage 3 virus stock that was used as the exposure virus for the NHP experiments. The cells and virus were those chosen by the Filovirus Animal Non-clinical Group (FANG) for use in near-term animal studies. The FANG recommended that virus used in animal exposure studies be low passage, from a lethal human case, and be well characterized (including genomic sequencing, sterility, mycoplasma, and endotoxin testing) [27]. Importantly, to maximize efficiency of animal biosafety level 4 (ABSL-4) studies, selection of a single variant generated from the same isolate may permit direct comparisons of data between the limited number of facilities performing advanced countermeasure testing. At the time this study was performed, an appropriately well-characterized stock of EBOV Makona variant was not available.

### 2.3. Determination of Viral Titers

Viral titers were determined by plaque assay using Vero E6 cells and agarose overlay as previously described [28].

### 2.4. Effect of LMA^®^ MAD on Ebola Virus Infectivity

To determine if delivery via the LMA^®^ MAD affected EBOV infectivity, virus was diluted in MEM and 300 µL were loaded into a 1 mL syringe. This material was expressed through the LMA^®^ MAD into MEM and care was taken to place the tip of the MAD below the surface of the media to capture all of the expressed material. The amount of infectious virus (plaque forming units per mL; PFU) captured in the media was determined by plaque assay and compared to the titer of the non-expressed starting material. The plaque assay was performed in duplicate and the experiment repeated four times.

### 2.5. Animal Studies

Research naïve animals were acquired from Covance (Vietnamese origin) and screened by Virus Reference Laboraty (VRL, San Antonio, TX, USA) for serum reactivity to Reston virus nucleoprotein. Animals with no sero-reactivity were shipped to TBRI and underwent a standard quarantine period. During quarantine, animals acclimated to single housed caging and in-house diet. Animals were monitored at least twice daily and enrichment included commercial toys and food supplements. Prior to blood collections, animals were anesthetized using Telazol (Zoetis Inc., Parsippany-Troy Hills, NJ, USA) [25,26]. Animals were randomly assigned to groups. Eight male and three female cynomolgus macaques (*Macaca fascicularis)*, 3.5–7 years of age, 2.5–6.5 kg in weight, were used for this report. For each study, EBOV exposure occurred on study day zero. Test subjects were then observed at least twice daily for up to 11 days post exposure (p.e.). Critically ill animals were observed at least four times per day and euthanasia criteria specific to NHPs and the exposure agent were followed to minimize pain and distress.

### 2.6. Experimental Inoculation of NHPs with EBOV

Four male *M. fascicularis* were injected IM in the deltoid muscle with a target dose of 100 PFU of EBOV (back titer indicated the dose was 74 PFU). Blood samples were collected on days 0, 3, 5, and 7 p.e. and at time of euthanasia. For IN group A, four *M. fascicularis*, two female and two male, were exposed IN using the LMA^®^ MAD with a target dose of 120 PFU of EBOV (back titer indicated the dose was 80 PFU). Blood samples were collected on days 0, 3, 5, 7 p.e. and at time of euthanasia. For IN group B, three *M. fascicularis*, one female and two male, were exposed IN using the LMA^®^ MAD with a target dose of 500 PFU of EBOV (back titer indicated the dose was 64 PFU). Blood samples were collected on days 0, 3, 6, 9 p.e. and at time of euthanasia. During all blood collections, rectal temperature and weight were recorded.

### 2.7. Determination of Viral Genome Copies

Quantitative reverse transcription-PCR (RT-PCR) was used to determine the number of viral genomes present in the serum of animal 36146 (IN group A) due to levels of cell culture infectious virus being below the limit of detection, as determined by plaque assay. An aliquot of serum was diluted in RNAbee (Tel-Test, Friendswood, TX, USA) after collection and transferred to the biosafety level 2 (BSL2) laboratory where RNA was harvested according to the manufacturer's instructions. Quantitative RT-PCR was performed as previously described [24] using primers and probe specifically designed to detect a region of the glycoprotein gene. The assay was run on an Applied Biosystems 7500 real-time PCR instrument using the following cycling conditions: 50 °C for 15 min (1 cycle), 95 °C for 5 min (1 cycle), 95 °C for 1 s and 60 °C for 35 s (45 cycles), and 40 °C for 60 s (1 cycle). A single fluorescence read was taken at the end of each 60 °C step [25].

### 2.8. Hematology, Coagulation and Blood Chemistry

Biochemical analysis was performed using the mammalian liver enzyme profile rotor on a Vet Scan analyzer (Abaxis, Inc., Union City, CA, USA). Coagulation times were determined using the IDEXX Coag Dx Analyzer (IDEXX Laboratories, Westbrook, ME, USA). Vetscan HM2 hematology analyzer (Abaxis, Inc., Union City, CA, USA) (IACUC 1381) or a Procyte Dx Hematology Analyzer (IDEXX laboratories, Westbrook, ME, USA) (IACUCs 1532 and 1529) was used to obtain neutrophil, lymphocyte, and red blood cell counts.

### 2.9. Statistics

The log-rank Mantel-Cox test was used to analyze the survival curves and the *p* value is displayed on each graph. GraphPad Prism two-way ANOVA was used to analyze blood chemistry.

## 3. Results

### 3.1. Effect of LMA^®^ MAD on Ebola Virus Infectivity

To determine if delivery via the LMA^®^ MAD affected EBOV infectivity, virus was diluted in minimum essential media (MEM) and loaded into a 1 mL syringe. The material was expressed through the LMA^®^ MAD into MEM and the amount of infectious virus (PFU) captured in the media was determined by plaque assay and compared to the titer of the starting material. Overall, there was a modest impact on infectivity. Material sprayed through the device contained an average of 2.8 fold less PFU than the starting material prior to spray (*n* = 4). It was decided not to correct for this effect when preparing the exposure dose to be administered to NHPs.

### 3.2. Survival and Viremia in NHPs Exposed to EBOV via IN or IM Route

To characterize the outcome of IN exposure with EBOV in NHPs, seven cynomolgus macaques were given a target dose of 120 PFU (*n* = 4, group A) or 500 PFU (*n* = 3, group B) via IN route using the LMA^®^ MAD (Table 1). For comparison to IM exposure, four NHPs were exposed via IM route with 100 PFU of EBOV (shown previously to be 100% lethal at this dose [25,26]).

**Figure 1:**
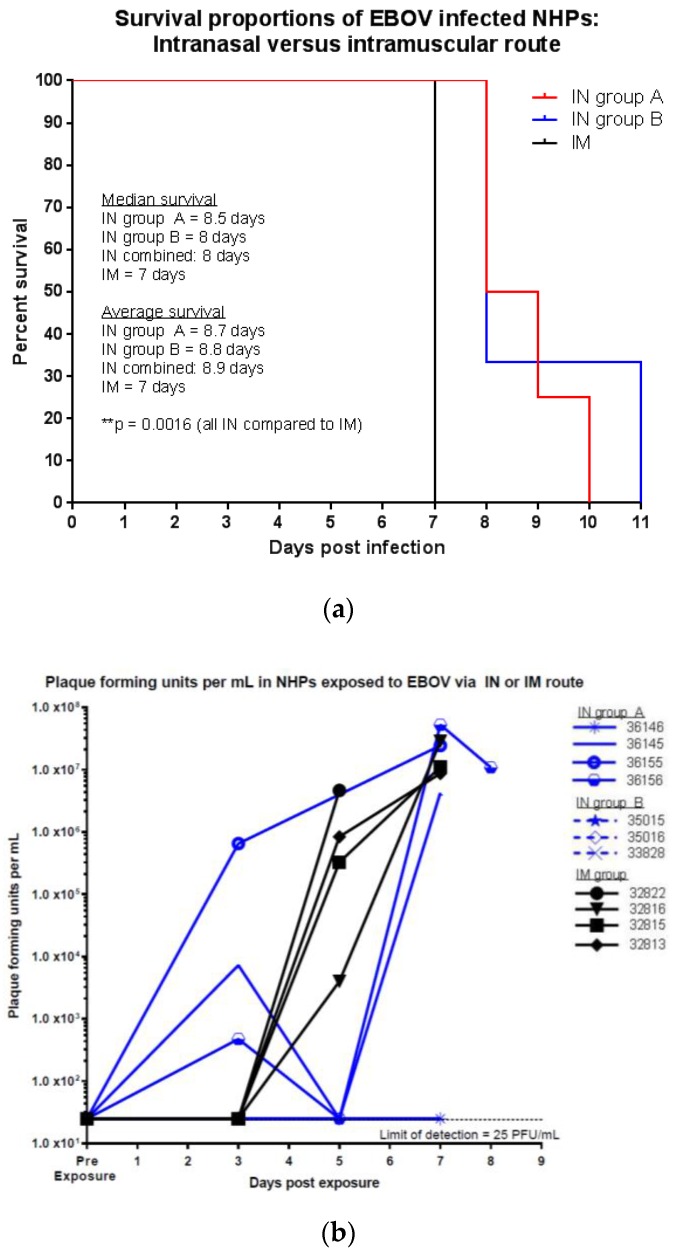
Survival and infectious virus titers in EBOV exposed NHPs (**a**) survival proportions of NHPs exposed to EBOV via IM or IN route, ** *p* ≤0.01; (**b**) plaque forming units of EBOV per mL in serum of NHPs exposed via IM or IN route.

As shown in Figure 1a, in the IN exposed animals, the time from exposure to death was significantly longer compared to the IM exposure group (*p* = 0.0016, Log-rank (Mantel-Cox), median 8 days and average 8.9 days compared to median 7 days and average 7 days). There was no significant difference in time to death from exposure between IN groups A and B (*p* = 0.5766).

Viremia was determined by plaque assay (Figure 1b). Serum samples were taken from each animal at scheduled time points. On day 3 post exposure (p.e.), three of seven animals exposed via IN and none of the animals exposed via IM exhibited measurable levels of infectious virus in the serum. On day 5 p.e., two of four animals exposed via IN (group A) and all animals exposed via IM exhibited measurable levels of infectious virus in the serum. Animals exposed via IN in group B were bled on day 6 p.e. and two of three exhibited measurable levels of infectious virus in the serum. On or near the day of death, six of seven animals exposed via IN and all animals exposed via IM exhibited high levels of infectious virus in the serum. However, one animal exposed via IN (group A) that succumbed on day 10 p.e. did not exhibit measureable levels of infectious virus in the serum throughout the course of the study. Due to the lack of detectable levels of infectious virus in the serum of this animal, quantitative reverse transcription-PCR (RT-PCR) was used to quantify the viral genome equivalents (GEs) present in the serum and to verify that the animal had become infected with EBOV after exposure. Viral RNA was detected by RT-PCR on days 5 (5.95 × 10^5^ GEs/mL) and 7 (2.58 × 10^7^ GEs/mL) p.e., indicating the animal was infected and viremic but not at levels sufficient for detection via plaque assay.

### 3.3. Other Clinical Parameters in NHPs Exposed to EBOV via IN or IM Route

**Figure 2:**
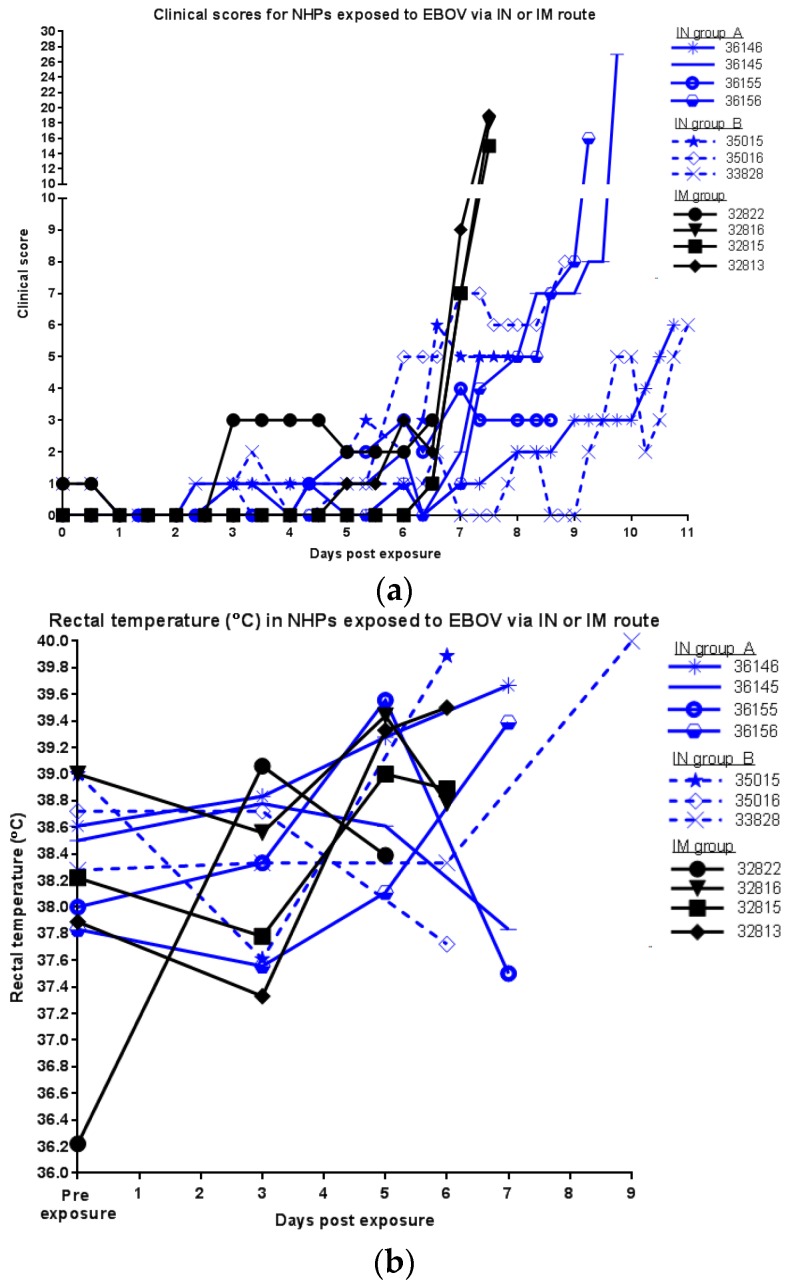

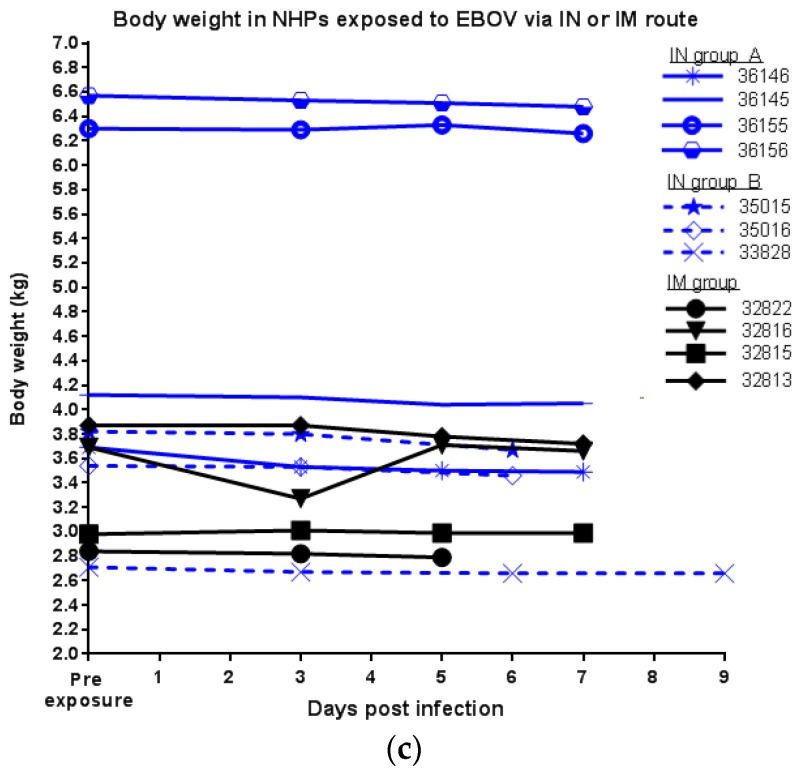
Clinical observations of EBOV infected NHPs exposed via IM or IN route; (**a**) clinical scores; (**b**) rectal temperature (°C); (**c**) body weight (kg).

Animals were observed at least twice daily and clinical observations were recorded (Figure 2a). Early signs of decreased food consumption (less than 25% feed consumed), reduced fluid intake, and reduced stool production were observed by day 3 p.e. Clinical signs indicative of EBOV exposure in NHPs (increased rectal temperature, petechia, and decreased responsiveness) were observed in some animals starting on days 5 to 6 p.e., but IN exposed animals were delayed in exhibiting increased scores when compared to the IM group. For animals exposed via IM route, clinical scores increased more substantially on day 7 p.e. with animals exhibiting petechia and dehydration, in addition to the previously noted reductions in feed consumption, fluid intake, and stool production. Animals also began exhibiting decreased responsiveness and withdrawn attitudes. All animals in this group succumbed to infection at this point, with two exhibiting rough hair coat and labored breathing in addition to the aforementioned symptoms. For animals exposed via IN route, animals began exhibiting petechia and dehydration on day 6 p.e., in addition to the previously noted reductions in feed consumption, fluid intake, and stool production. Reduced responsiveness appeared on days 7 and 8 p.e. However, unlike the IM group, these animals did not immediately succumb at this point. Rather, there was a day or two delay before most IN exposed animals exhibited substantially increased clinical scores and two animals did not exhibit more severe signs until days 10 and 11 p.e.

Another important parameter in the cynomolgus macaque model of EBOV infection is febrility (Figure 2b). As disease progresses, many animals become febrile (seen with many of the animals on day 5 p.e.) sometimes preceding a brief period of hypothermia prior to death. However, there were no differences in temperature between the exposure groups. Changes in body weight (Figure 2c) were insignificant throughout the course of the study and among the different exposure groups.

### 3.4. Hematology and Blood Chemistry in NHPs Exposed to EBOV via IN or IM Route

**Figure 3:**
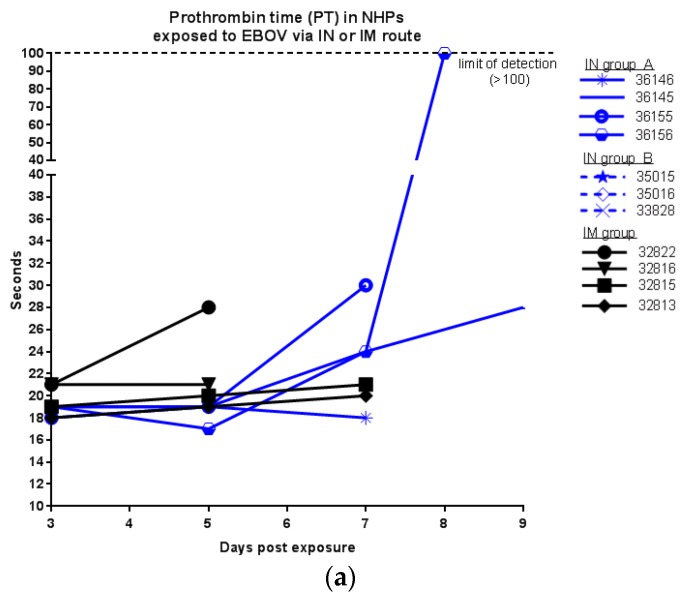

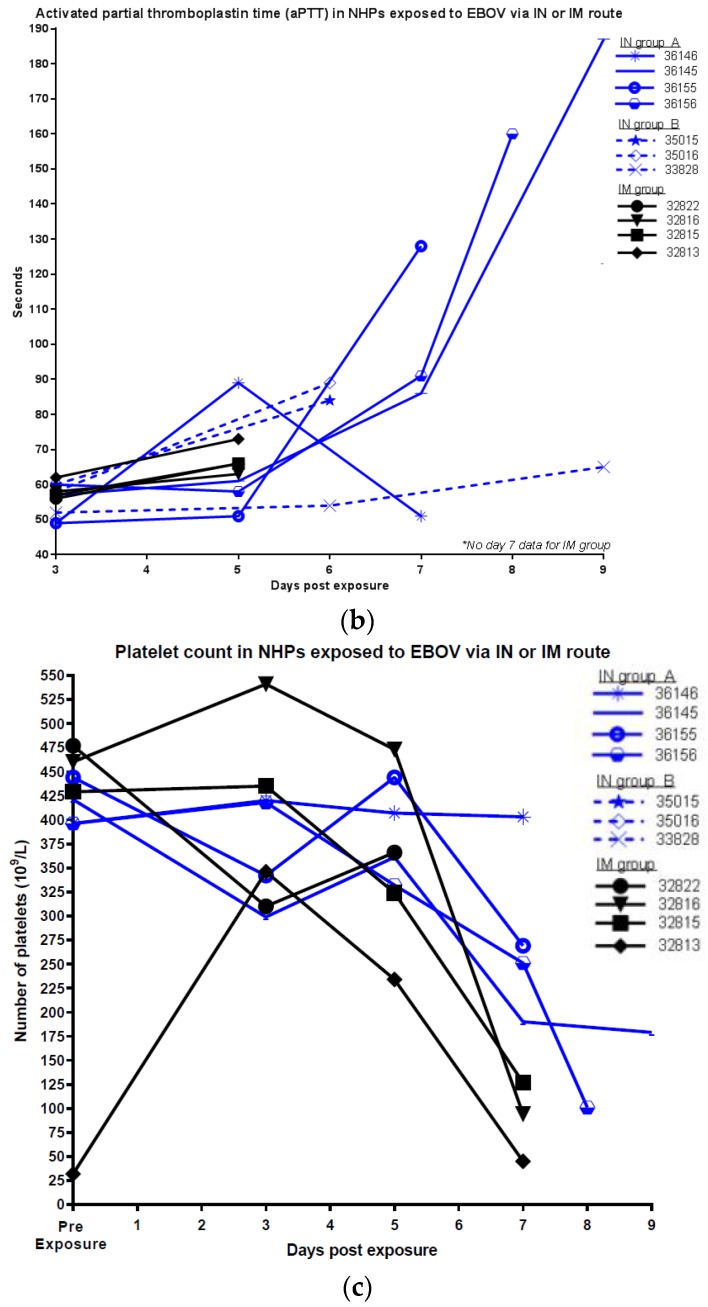
Coagulation tests of EBOV infected NHPs exposed via IM or IN route; (**a**) PT; (**b**) aPTT, *no day 7 data for IM group; (**c**) platelets.

Coagulation tests, complete blood counts, and chemistry analyses were performed on blood collected at designated time points during the study. Prolonged coagulation times were more pronounced in NHPs exposed via IN (Figure 3). Only one animal exposed via IM exhibited an increased prothrombin time (PT). While all IM animals exhibited slight increases in activated partial thromboplastin times (aPTT) at day 5 p.e., data are not available for day 7. Within the IN groups, six of seven animals exhibited prolonged PT and all animals exhibited prolonged aPTT (though animal aPTT in 36146 declined again on day 7 p.e.). All animals except for 36146 (IN group A) exhibited decreased platelets, generally after day 3 p.e. (Figure 3c) suggestive of disseminated intravascular coagulation.

**Figure 4:**
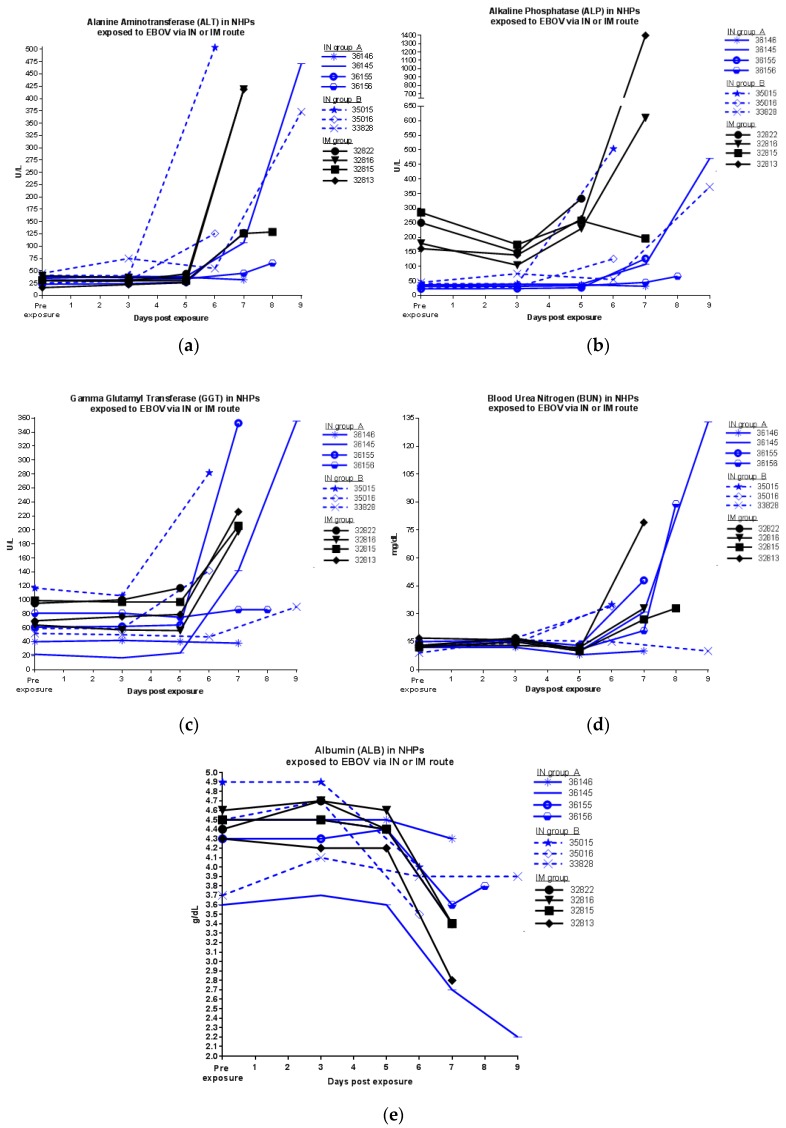
Blood chemistry of NHPs exposed to EBOV via IN or IM route; (**a**) ALT; (**b**) ALP; (**c**) GGT; (**d**) BUN; (**e**) ALB.

Clinical chemistry parameters showed shifts from pre-exposure levels (Figure 4). Increases in clinical chemistry parameters indicative of marked liver damage (increases in ALT, ALP, and GGT) were observed just prior to euthanasia for most animals. Increased BUN) and decreased albumin were also noted (Figure 4). Most shifts in clinical parameters were similar between IN and IM exposure groups. However, animals exposed via IN exhibited less dramatic increases in ALP and were delayed in exhibiting increased values. Exposure route was significantly responsible for this variation in ALP (*p* = 0.002, Two-way ANOVA comparing IM group to IN group A; IN group B cannot be included in Two-way ANOVA analysis because blood sampling time points were different). Exposure route also correlated with a significant difference in ALT on day 7 p.e. (*p* = 0.0405, Two-way ANOVA comparing IM group to IN group A, with animal 32822 excluded; this animal was found dead on day 7 and thus no blood sample was available for comparison at this time point).

**Figure 5:**
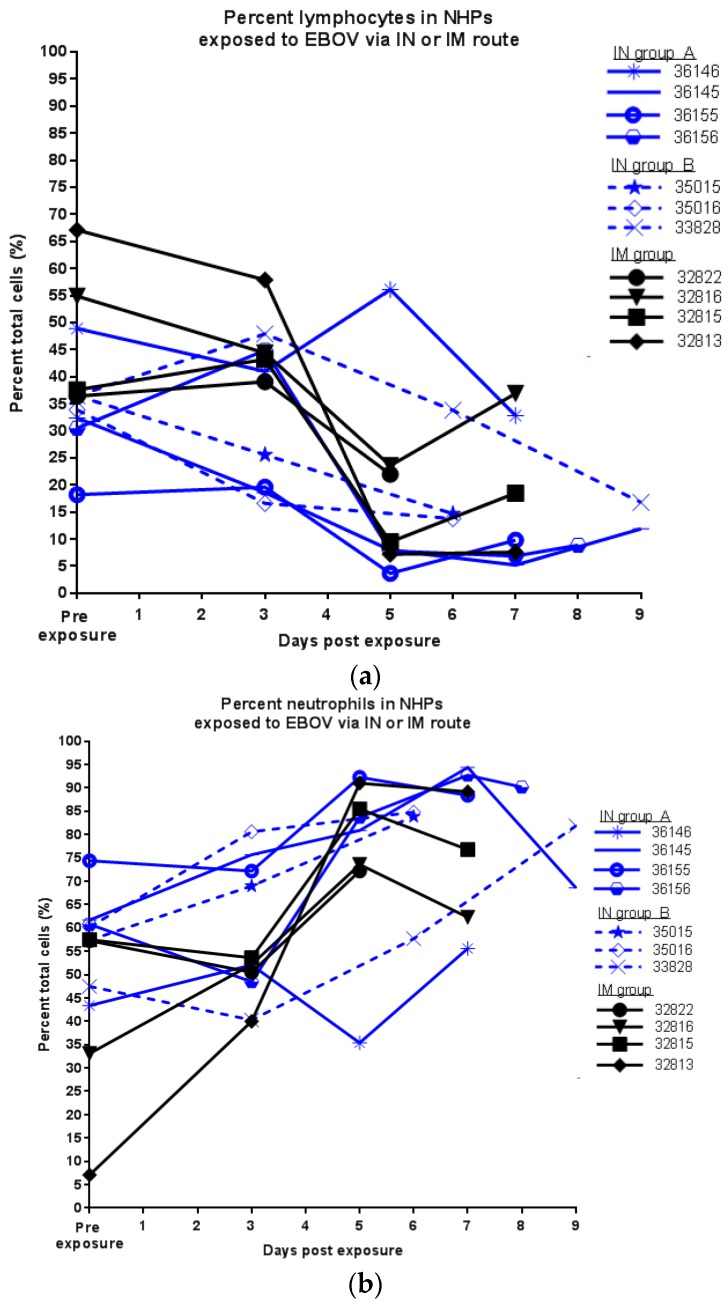
Lymphocytes and neutrophils in NHPs exposed to EBOV via IN or IM routes; (**a**) percentage of lymphocytes; (**b**) percentage of neutrophils.

Animals in both groups exhibited increases in levels of neutrophils and decreases in levels of lymphocytes (Figure 5), also indicative of disease in the cynomolgus macaque model of EBOV infection [25,26].

Animals in both exposure groups exhibited red blood cell (RBC) counts there were below the normal range. However, animals exposed via IM exhibited decreased counts on day 3 p.e. (animals 32813 and 32822) while animals exposed via IN did not begin exhibiting decreases until days 5 p.e. (36155, IN group A) and 6 p.e. (35016 and 33828, IN group B). Within IN group A, 36145 and 36155 also exhibited low counts on day 7 p.e. and animal 36156 exhibited low counts on day 8 p.e. Three animals exposed via IM also exhibited RBC counts below the normal range on day 7 p.e. (animal 32822 was found dead on day 7 and thus no blood sample was available, but the animals had values below the normal range on day 5 p.e.).

### 3.5. Pathology in NHPs Exposed to EBOV via IN or IM Route

Gross pathology findings for both exposure routes are summarized in Table 2. Within NHPs exposed by either route, significant gross findings included: petechia, testicular hemorrhage, and dark and red lungs. As expected, pathology was observed in the lymph nodes nearest the site of exposure. In the IM group, edema was observed surrounding the right axillary lymph node (IM exposure occurred in right deltoid) in two of four NHPs. Within the IN groups, the following was observed: dark and red submandibular lymph nodes in two of seven NHPs and dark, enlarged mediastinal lymph nodes in five of seven NHPs. Within the IN groups, lungs were described as dark and red in five of seven NHPs with adhesions also found in one NHP. No abnormalities were noted at the exposure site (nares). Within the IM group, lungs were described as dark and red in three of four animals. Animals exposed via IN route had a higher incidence of findings within the gastrointestinal (GI) tract (e.g., red and or dark red areas within the GI tract).

Significant histologic findings are summarized in Table 3. Findings for all groups were consistent with acute filoviral infection and included: lymphoid depletion and necrosis; splenic fibrin deposition; and hepatocellular necrosis. Within the IN groups, no findings were noted at the challenge site (nares) and pulmonary changes were generally mild and consisted of edema, inflammation, necrosis, and hemorrhage. Consistent with macroscopic findings, many animals exposed via IN route exhibited evidence of GI hemorrhage.

## 4. Discussion

No approved vaccines or therapies exist for filovirus infections and there is need for advancements in this area. The development and testing of effective countermeasures against EBOV requires well-characterized animal models and a thorough understanding of those models. However, in the majority of existing models, the most common exposure route has been parenteral [13,17]. Further work is needed to develop and characterize models that represent the bodily-fluid transmission seen during human outbreaks [7,14,29,30].

To characterize the outcome of IN exposure with EBOV in NHPs, seven cynomolgus macaques were exposed to EBOV via IN route using the LMA^®^ MAD. Exposure via the IN route allows for immediate contact with mucosal membranes and the potential for rapid infection of mononuclear phagocytic cells, which are thought to be the primary site of infection [31,32]. For comparison to IM exposure, four NHPs were exposed to EBOV via IM route. The virus used for all exposures was the Kikwit variant of Ebola virus, based on guidance from FANG [27]. Further studies are needed to determine how the Kikwit variant compares to the Makona variants involved in the western Africa outbreak, though recent comparisons in NHPs have suggested differences [19,33].

For the IN exposed animals, there was a statistically significant delay in the time-to-death after exposure compared to the IM exposed animals. In addition, animals exposed via IN exhibited a delay in developing high clinical scores post-exposure when compared to the IM group. For animals exposed via IM route, clinical scores increased more substantially on day 7 p.e. with animals exhibiting decreased responsiveness and rapid progression to death. For animals exposed via IN route, animals also began exhibiting reduced responsiveness on days 7–8 p.e. However, disease in these animals did not progress as rapidly and they died at a later time than the IM infected animals.

This increase in the time to death from exposure is consistent with other studies testing mucosal exposure routes including oral and ocular exposures [18]). The longer time to death after IN exposure in the NHP model more closely aligns with that observed in human infections and the current 21 day quarantine/monitoring period for personnel returning from the front line of an outbreak [34,35]. Moreover, the higher frequency of GI pathology seen within the IN groups, compared to the IM group, may suggest more GI involvement after exposure via the IN route. Gastrointestinal symptoms are a common manifestation of EVD in humans [34,35], but the IM exposed NHP models do not often recapitulate this.

Most clinical parameters were similar between IN and IM exposure routes (e.g., rectal temperature, body weight, GGT, BUN, ALB). However, some markers of liver damage were more pronounced in the IM group; ALT on day 7 p.e. and ALP were significantly different between the two exposure routes. Furthermore, viremia was more variable within the IN groups than in the IM group, which did not exhibit measurable levels of infectious virus in the serum until day 5 p.e. but then viremia increased at or near time of death. Three animals from IN group A exhibited measureable levels of infectious virus in the serum on day 3 p.e., with one animal (36155) exhibiting 3.9 × 10^5^ PFU/mL. Titers from serum collected during subsequent time points were less consistent than those observed with the IM group, with infectious virus waning at times rather than steadily increasing. Furthermore, one animal (IN group A) succumbed on day 10 p.e. without exhibiting measureable levels of infectious virus throughout the course of the study, though viral RNA was detected on days 5 and 7 p.e. (this may be related to the high genome to PFU ratio often seen with filoviruses). The differences seen in liver values (ALP and ALT) and serum viremia between IN and IM exposed NHPs may point to variable sites of viral replication during exposure via different routes.

Much remains unknown about natural spread of EBOV and how exposure route affects disease course. Furthermore, it is often difficult or impossible to discern when and how an individual became infected [7,36]. Animal models that represent various exposure routes can provide more information on these topics. A well-characterized IN NHP model can offer insight into the consequences of exposure to bodily fluids via a mucosal surface, such as the nasal epithelium. For example, the delay in time to death after IN exposure may help elucidate the differences in survival seen during human infections. Moreover, further work is also needed to optimize strategies for preventing spread of infection during outbreaks, especially in remote areas [37]. Models of mucosal exposure will be valuable tools for such work. In addition, countermeasures may need to be screened via different exposure routes, especially given information learned about EBOV transmission during human outbreaks. It is likely that exposure to bodily fluids via a mucosal route plays an important role in human outbreaks [7,14,16] and further information is needed to determine how exposure route impacts viremia, sequelae, immune response, and overall outcome. Furthermore, the frequently used IM exposure route was developed to model laboratory exposures and screening countermeasures first via the IM route may be too stringent. Utilizing an alternative, relevant route may be crucial for countermeasure screening and development. For example, models of alternative exposure routes may be valuable in informing future vaccination strategies. Efficacy of IM vaccinations can be evaluated using a mucosal exposure model and vice versa. Thus, an IN model of exposure offers a useful alternative to parenteral models as it can offer insight into the consequences of exposure via a mucosal surface and offers a realistic and relevant comparison to human EBOV infections.

## Figures and Tables

**Table 1:** Study design.

Group	Exposure Route	Number of Animals	Target Dose for EBOV Exposure [PFU]	Determined Dose for EBOV Exposure [PFU]
IN group A	intranasal	4	120	80
IN group B	intranasal	3	500	64
IM	intramuscular	4	100	74

**Table 2:** Summary of macroscopic findings.

Group	Petechial Rash	Testicular Hemorrhage	Dark, Red Axillary Lymph Node	Pale or Enlarged Spleen	Pale, Friable Liver	Dark, Red Lungs	Redness or Hemorrhage in GI Tract
IN (A)	3/4	2/2	1/4	1/4	3/4	3/4	2/4
IN (B)	3/3	2/2	2/3	2/3	0/3	2/3	3/3
IM	4/4	2/4	1/4	1/4	0/4	3/4	1/4

**Table 3:** Summary of histopathology findings.

Organ	Histopathology Finding	Group (Number Exhibiting/Number in Group)		
		IM	IN A	IN B
Spleen	Fibrin deposition	3/4	4/4	3/3
	Lymphoid depletion	3/4	4/4	3/3
	Lymphocytolysis	3/4	4/4	3/3
	Congestion/hemorrhage, marginal sinus	2/4	4/4	3/3
Lymph node	Lymphoid depletion	3/4	4/4	3/3
	Necrosis	4/4	4/4	3/3
Liver	Hepatocellular necrosis	2/4	4/4	3/3
Lung	Edema	n.d.	2/4	2/3
	Fibrin deposition	n.d.	2/4	0/3
	Inflammation	n.d.	3/4	1/3
	Necrosis	n.d.	1/4	1/3
	Hemorrhage	n.d.	2/4	1/3
				
Adrenal gland	Individual cell necrosis	0/4	3/4	3/3
Testicle	Hemorrhage	3/4	2/2	2/2
				
GI Tract	Hemorrhage	1/4	2/4	2/3

n.d.—no data available.
